# 41^st^ Annual Conference

**DOI:** 10.4103/0253-7613.45146

**Published:** 2008

**Authors:** Shiv Prakash

**Affiliations:** Chief Editor

I wish all the participants of the 41^st^ Annual Conference of the Indian Pharmacology Society a great success in all their scientific endeavors. This time, it's being held at AIIMS, New Delhi. The conference also coincides with the International Conference on Translational Pharmacology. It's time to meet peer groups, friends, and renewing lost contacts.

I take this opportunity to thank all the Executive Committee members who have worked very hard throughout the year to make important decisions for the Society. Some of them are historic, for example, it was decided to bring a white paper on the development of pharmacology in India with a special focus on how it can be further developed to be in sync with international research. This will showcase the contributions made by all the senior pharmacologists of India and may serve as a great record to preserve their contributions.

There has been a consensus on putting into place a standard operating procedure on various prizes which are to be awarded during the Annual Conference. This will help future members take decisions with greater transparency and serve as a great confidence builder for all the participants in competitions.

An important discussion that needs to take place in this General Body meeting is to find a permanent home for the Pharmacology Society. The conference is being held in Delhi which may be accepted as the home for our Society.

I would like to again thank Dr. Jagadeesh, USFDA for conducting the preConference workshop on scientific writing. Every year, his efforts have a profound effect on young pharmacologists as the topics covered are important and updated. Like last year, this workshop's deliberations will be published in the Indian Journal of Pharmacology for the benefit of those who missed the opportunity to attend this excellent event.

I welcome the new incumbent, Prof. R. K. Goyal, Vice Chancellor, Baroda University, as the new President of the Society next year. He takes over from the veteran pharmacologist, Prof. Manjeet Singh and will bring many new perspectives to the Society. Prof. Goyal has served the Society in various capacities and his great networking capability would bring professionals closer.

It takes lot of hard work, time, and personal commitment to conduct the conference. I appreciate the pains taken by the Organizing Committee of AIIMS in organizing this Annual Conference. For the first time, the Abstracts supplement of the journal will be available during the inaugural session.

I cannot forget my Editorial team and publisher. They have done a tremendous job to make the journal a great place to publish pharmacology research. I thank Medknow Publications for their support in bringing fresh design covers every time.

I wish all our readers a very happy and prosperous new year.

